# Opening of the New Italian Hospital

**Published:** 1900-03-17

**Authors:** 


### OPENING OF THE NEW ITALIAN HOSPITAL.

The handsome and admirably appointed new buildings of the Italian Hospital in Queen Square were opened by the Italian Ambassador on Wednesday afternoon, the day being chosen because it was the anniversary of the birthday of the King of Italy. The late Commendatore Ortelli, who gave the original building, provided for the extension before his death, and the noble benefaction represents a gift of over £23,000. His widow presented the Italian Ambassador with a gold key of the hospital, who having declared it open, presented Mrs. Ortelli with a magnificent gold jewelled medallion of King Humbert from himself, with a special inscription in memory of her husband, and in recognition of his generosity.

